# PARP inhibitor resistance in IDH1-mutant cancers due to loss of end protection factors, 53BP1 and REV7

**DOI:** 10.1093/narcan/zcaf047

**Published:** 2025-12-03

**Authors:** Daniel Andrés Colón-Ríos, Jonathan Dow, Adam Krysztofiak, Yanfeng Liu, Faye A Rogers, Peter M Glazer

**Affiliations:** Department of Genetics, Yale School of Medicine, New Haven, CT 06510, United States; Department of Therapeutic Radiology, Yale School of Medicine, New Haven, CT 06510, United States; Department of Genetics, Yale School of Medicine, New Haven, CT 06510, United States; Department of Therapeutic Radiology, Yale School of Medicine, New Haven, CT 06510, United States; Department of Therapeutic Radiology, Yale School of Medicine, New Haven, CT 06510, United States; Department of Therapeutic Radiology, Yale School of Medicine, New Haven, CT 06510, United States; Department of Therapeutic Radiology, Yale School of Medicine, New Haven, CT 06510, United States; Department of Genetics, Yale School of Medicine, New Haven, CT 06510, United States; Department of Therapeutic Radiology, Yale School of Medicine, New Haven, CT 06510, United States

## Abstract

Acquired resistance presents a major challenge for targeted therapies, with initially responsive tumors eventually reverting underlying vulnerabilities. Our group recently reported that cancers harboring isocitrate dehydrogenase 1/2 (IDH1/2) mutations have defective recruitment of homology-directed repair (HDR) factors to sites of DNA damage and consequent sensitivity to poly(ADP-ribose) polymerase inhibitors (PARPi), a vulnerability that is being tested in clinical trials. To probe potential mechanisms by which resistance to PARPi might arise in this setting, we modeled PARPi resistance in IDH-mutant tumors via serial transplantation of patient-derived xenografts in mice treated with PARPi. An analysis of candidate DNA repair factors in these resistant tumor populations identified downregulation of two end protection factors that are negative regulators of HDR, 53BP1, and REV7. Knockout of these factors by CRISPR–Cas9 in IDH1-mutant cancer cells conferred robust resistance to PARPi and restored HDR capacity. To overcome this resistance, we found that treatment with the receptor tyrosine kinase inhibitor, cediranib, previously reported to suppress expression of downstream HDR factors, resensitizes 53BP1 and REV7-knockout cells to PARPi treatment. Our findings identify key pathways driving PARPi resistance in IDH1-mutant cancers and highlight potential therapeutic strategies to overcome this resistance.

## Graphical Abstract

**Graphical Abstract:**
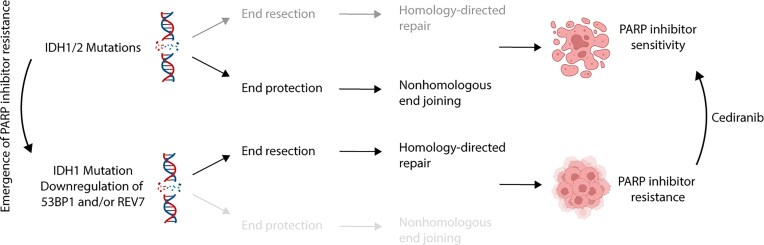

## Introduction

Mutations in the isocitrate dehydrogenase 1 and 2 (IDH1/2) genes are found in 80% of low-grade gliomas, 20% of secondary glioblastomas, 20% of acute myeloid leukemias, 20% of cholangiocarcinomas, and 80% of chondrosarcomas, among other malignancies [1]. Most of these IDH1/2 alterations are heterozygous missense mutations that confer a neomorphic activity on the encoded enzymes, such that they convert α-ketoglutarate to (R)-2-hydroxyglutarate (2HG), identified as an oncometabolite that contributes to the malignant phenotype in these cancers [1]. Based on this, one prominent therapeutic strategy has been to advance small molecule inhibitors of the neomorphic enzymes into the clinic [1]. Further characterization of IDH-mutant cancer cells by our group and others led to the discovery that 2HG causes a DNA repair defect through alteration of DNA repair factor recruitment to sites of DNA damage, conferring vulnerability to DNA repair inhibitors, including poly (ADP-ribose) polymerase inhibitors (PARPi) [2–4].

When a double strand break (DSB) occurs in healthy cells, the methyltransferase SUV39H1 methylates H3K9 around the site of damage, initiating a cascade that includes recruitment of homology directed repair (HDR) factors TIP60 and MRE11 [5]. Our group previously demonstrated that 2HG inhibits the lysine demethylase KDM4B, leading to aberrant and excessive H3K9 trimethylation (H3K9me3) that compromises the normal chromatin signaling at H3K9 to initiate DNA repair [3], as elevated H3K9me3 levels mask the H3K9me3 spike that occurs in normal cells, impairing recruitment of HDR proteins. 2HG also impacts DNA repair by inhibiting the catalytic activity of ten-eleven translocation methylcytosine dioxygenases (TET1/2) enzymes, leading to genome-wide DNA hypermethylation. This hypermethylation phenotype can affect the binding of methylation-sensitive factors such as CCCTC-binding factor (CTCF), which has been reported to be involved in HDR by recruiting BRCA2 to sites of DSB and interacting with RAD51 to promote repair [6, 7]. Indeed, it was recently reported that 2HG suppresses CTCF binding, thereby impairing the assembly of HDR DNA repair complex at sites of DSBs [8]. Together, these defects in HDR render IDH-mutant cancers sensitive to PARPi. In addition, IDH-mutant cancer cells also show heterochromatin-related replication stress, further contributing to PARPi sensitivity [9], in a mechanism that would require HDR to resolve PARPi-induced breaks at replication forks [10].

Over the last decade, PARPi have been used as monotherapy and in combination with other therapeutics for the treatment of other HDR-deficient cancers, most notably BRCA1/2-mutated cancers. Initial trials in ovarian cancer demonstrated efficacy leading to FDA-approval therapy [11–13]. Nonetheless, many patients with BRCA1/2-mutant cancers treated with PARPi will develop resistance to treatment [14, 15]. In patients and experimental models, resistance has been shown to arise via multiple mechanisms, including reversion mutations [16, 17], restoration of HDR [18–25], and replication fork stabilization [26, 27], among others [28]. Since PARPi have been only recently implemented in clinical trials for patients with IDH-mutant cancers [29], the potential mechanisms of PARPi resistance in this context remain undefined, highlighting the need for further studies to elucidate possible resistance pathways and to potentially guide patient selection, trial design, and new therapeutic strategies.

Here, we describe loss of end protection factors 53BP1 and REV7 as a mechanism of PARPi resistance in IDH1-mutant cancers. We conducted serial xenograft tumor studies with patient-derived IDH1-mutant HT1080 fibrosarcoma cells, isolating tumor populations exhibiting resistance to PARPi treatment. In these resistant cell populations, we detected downregulation of the DNA end protection factors REV7 and 53BP1 that regulate pathway choice in repair of DNA DSBs [30, 31]. Directed knockout of these genes in HT1080 cells not only led to PARPi resistance but also led to restoration of HDR and an overall decrease in baseline DNA damage. As a strategy to overcome this resistance, we leveraged our previous observation that cediranib, a receptor tyrosine kinase inhibitor, decreases HDR through inhibition of platelet-derived growth factor receptor β (PDGFRβ), activation of phosphatase PP2A, formation of repressive E2F4/p130 complexes, and consequent downregulation of HDR gene expression [32]. We find that treatment of cells that have PARPi resistance due to loss of 53BP1 and REV7 with cediranib can overcome the resistance and resensitize cells to PARPi. These findings provide mechanistic insight into potential PARPi resistance in IDH-mutant tumors and identify cediranib as a potential agent to overcome PARPi resistance.

## Materials and methods

### Cell culture

HT1080 cells (ATCC) were cultured in Dulbecco’s Modified Eagle Medium (DMEM) containing 10% fetal bovine serum (FBS, Gibco). U2OS EJ-DR cells were obtained from Ranjit Bindra (Yale University School of Medicine) and were maintained in DMEM + 10% FBS. HCT116 IDH1+/+ and HCT116 IDH1+/R132H (ATCC) were cultured in McCoy’s Media + 10% FBS. Cells were passaged at least twice after thawing before use in experiments and were regularly tested for *Mycoplasma* (MycoAlert, Lonza).

### Drugs, chemicals, and reagents

Talazoparib, olaparib, and cediranib were obtained from SelleckChem and dissolved in dimethyl sulfoxide (DMSO, Sigma-Aldrich) for cell culture experiments. (2R)-octyl-α-hydroxyglutarate was obtained from Cayman Chemical and dissolved in DMSO. For animal tumor studies, talazoparib was diluted in a vehicle containing 10% dimethylacetamide, 6% Kolliphor HS 15 and 84% PBS, and administered to mice via oral gavage 5 days a week, for 3 weeks. Horizon Dharmacon ON-TARGETplus HUMAN SMARTpool siRNAs targeting TP53BP1 (53BP1), MAD2L2 (REV7), and a nontargeting control pool were resuspended in water and used at 20 nM. Horizon Dharmacon single-guide RNAs (sgRNAs) targeting TP53BP1, MAD2L2 and a nontargeting control were resuspended in Tris Buffer pH 7.4 (Dharmacon Cat No B-006000-100). A full list of sgRNA sequences is presented in Supplementary Table S1.

### Generating PARPi-resistant HT1080 xenograft tumors

Human HT1080 (5 × 10^6^) were implanted subcutaneously in the flanks of athymic nu/nu mice (Harlan). Three times a week, tumors were measured using calipers to calculate tumor volume [*V* = ½(4π/3)(length/2)(width/2)(height)]. Tumors were treated with 0.33 mg/kg talazoparib 5 days after injection, following a 5 days on, 2 days off regimen. Once tumors reached ~600 mm^3^ tumor volume (∼16 days of treatment), the three largest HT1080 tumors were dissociated and pooled together to generate the R-HT cell line. This cell line was kept in culture under 25 nM talazoparib treatment before re-implanting subcutaneously in the flanks of athymic nu/nu mice. Mice were again treated with 0.33 mg/kg talazoparib for 20 days, and the two largest tumors (501 and 504) were individually dissociated and kept in culture under 25 nM talazoparib treatment. Animal protocol (IACUC number 2025-07 902) was approved by the Yale University Institutional Animal Care and Use Committee.

### Tumor cell dissociation

Tumors were dissected and manually dissociated in DMEM + 10% FBS with dispase (5 µg/ml) and collagenase (5 mg/ml) (Stem Cell Technologies). Tumor cells were then incubated at 37°C for 90 min, washed with 1× Dulbecco's phosphate-buffered saline (PBS, Gibco), incubated in 0.25% Trypsin for 5 min at room temperature, and passed through a 70 μm filter. Red blood cells were removed with incubation on ice for 10 min in Ammonium Chloride Solution (Stem Cell Technologies). Mouse cells were removed, and human tumor cells isolated, using a Mouse Cell Depletion kit (Miltenyi Biotec).

### HT1080 and HT1080 REV7 KO mouse xenograft studies

Human HT1080 and HT1080 REV7 KO (5 × 10^6^) were implanted subcutaneously in the flanks of athymic nu/nu mice (Harlan). Three times a week, tumors were measured using calipers to calculate tumor volume [*V* = ½(4π/3)(length/2)(width/2)(height)]. Mice were treated with 0.33 mg/kg talazoparib 5 days per week with a 2 day break, starting at tumor volumes of 100 mm^3^. Protocol (Protocol Number 2025-07 902) was approved by the Yale University Institutional Animal Care and Use Committee.

### Short-term cell viability assay

Short-term cell viability assays were performed using the luminescence-based CellTiter-Glo assay (Promega). One thousand cells/well were seeded in 96-well plates and treated with indicated drugs for a total of 6 days. Luminescence was quantified using a BioTek Synergy H1 Microplate Reader and signal was normalized to untreated control samples.

### Long-term cell viability assay

One thousand HT1080 and REV7 KO cells were seeded in 6-well plates. 16–24 hours after seeding, cells were treated with the indicated doses of PARPi. Cells were allowed to grow for a maximum of 14 days. Halfway through treatment (day 5), drug-containing media was removed, and cells were supplemented with drug-free media. Five hundred HCT116 cells and one thousand HCT116 IDH1 +/R132H cells were seeded in 6-well plates and left to attach for 16–24 h. The HCT116 match pair was treated with increasing doses of cediranib at the indicated doses and allowed to grow for 14 days, removing drugged media at day 5 of treatment and supplementing with drug-free media. Cells were fixed with 0.9% NaCl for 5 min and stained with crystal violet for at least 20 min.

### Intracellular D-2-hydroxyglutarate measurements

The D-2-hydroxyglutarate (D-2HG) assay kit (Abcam, ab211070) was used to measure intracellular levels of D-2HG in the HT1080 tumor populations according to the manufacturer’s protocol.

### Western blot

Whole-cell lysates were prepared from floating and adherent cells using AZ lysis buffer. Ice-cold lysis buffer containing 50 mM Tris pH 7.4, 250 mM NaCl, 1% IGEPAL, 0.1% SDS, 5 mM EDTA pH 8, 10 mM Na_2_P_2_O_7_, 10 mM NaF, 2 mM Na_3_VO_4_, 1× phosSTOP, and 1× protease inhibitor cocktail tablet was added to the cell pellets and incubated for 20 min on ice, vigorously mixing every 5 min. Samples were centrifuged at 16.1 × *g* for 10 minutes (4°C), supernatant (lysates) were extracted, and protein quantification was performed using Bio-Rad’s detergent-compatible protein assay (Cat No. 5000112). About 30–50 µg of total protein per sample were resolved by sodium dodecyl sulfate-polyacrylamide gel electrophoresis (SDS–PAGE). After transferring proteins onto a nitrocellulose membrane, the membranes were blocked for 1 h using 5% milk or bovine serum albumin in 1× Tris-buffer saline with 0.5% Tween-20 (TBST) and later immunoblotted using the primary antibodies listed in Supplementary Table S2.

### Knockout of 53BP1 and REV7

HT1080 knockouts were generated using Cas9 ribonucleoprotein complexes with three distinct sgRNAs. Briefly, three individual sgRNAs and SpCas9 (CP02, PNA Bio) were complexed at room temperature for 10 min in a 5 µl reaction at final concentrations of 0.5 µM/sgRNA and 0.45 µM, respectively. Immediately before nucleofection, Alt-R^™^ Cas9 Elec Enhancer (10007805, IDT) was spiked into the Cas9 RNP–sgRNA solution at a final concentration of 1 µM. For nucleofections, 1 × 10^6^ cells were resuspended in Lonza cell line solution. Cas9–sgRNA complex was added to the cells and nucleofected using a Lonza 4D-Nucleofector X unit. Cells were seeded in 2 ml of complete media. After expansion, pooled knockout populations were seeded at low density to select for clonal populations. Gene knockout was verified via western blot analysis.

A second REV7 knockout line was generated using the established lentiGuide-Puro/lentiCas9-Blast system [33]. HT1080 cells were engineered to stably express CRISPR–Cas9 using the lenti-Cas9 Blast plasmid (Addgene plasmid #52962). The CRISPR–Cas9-expressing HT1080 line was selected from blasticidin-resistant single-cell colonies based on CRISPR–Cas9 expression via western blot analysis. REV7 sgRNA (5′-GAGGTCTTGTCGTGTGAGCG-3′) was expressed in CRISPR–Cas9-expressing HT1080 line using the lentiGuide-Puro vector (Addgene plasmid #52963). Knockout was screened and confirmed from blasticidin and puromycin resistant single-cell colonies by expression via western blot analysis.

### EJ-DR HDR assay

U2OS EJ-DR cells were pretreated with siRNA for 3 days before adding 2HG and ligands, triamcinolone (100 nM, Sigma) and Shield1 (0.5 µM, Clontech), to media for 24 h. Ligands were washed, and cells were supplemented again with siRNA and 2HG. Cells were analyzed by flow cytometry after 3 days for GFP signal.

### Neutral comet assay

Cells were seeded in six-well plates at a density of 4 × 10^5^ cells per well 18 h prior to irradiation treatment, or at a density of 2.5 × 10^5^ cells per well prior to olaparib treatment. Four hours after treatment with 5 Gy, or 24 h after treatment with 1 µM olaparib, cells were collected, and the protocol was followed according to the manufacturer’s instructions (Trevigen, Inc.). Comet slides were lysed and electrophoresed for 1 h, stained with SYBR Gold^®^ and visualized using an EVOS^®^ FL cell imaging microscope. The data were analyzed manually with Comet Score 2.0^™^ software. Approximately 100–200 randomly selected nonoverlapping cells were analyzed per experiment. Experiments were performed at least three times and results are expressed as mean tail moment and standard error of the mean.

### Immunofluorescence

Cells (5 × 10^4^) were seeded in 8-chamber slides coated with collagen and irradiated 16–24 h later using an X-RAD 320 X-Ray Biological Irradiator (Precision X-Ray, Inc.). Cells were fixed and permeabilized with 3% paraformaldehyde, 0.5% Triton X-100, and 8% sucrose in 1× PBS for 15 min at room temperature and additionally fixed with ice-cold acetone:methanol (1:1) for 10 min at 4°C. Samples were incubated in blocking buffer (5% normal goat serum, 5% FBS, 0.5% Triton X-100, and 8% sucrose in 1× PBS) overnight at 4°C. Cells were incubated in RAD51 (1:400 dilution, PC130, EMD Millipore), BRCA1 (1:100 dilution, sc-6954, Santa Cruz), or phosphorylated RPA32 S4/8 (1:200 dilution, A300-245A, ThermoFisher) primary antibody for 1.5 h at room temperature, and overnight at 4°C. After three washes with 0.5% Triton X-100 and 8% sucrose in 1× PBS, samples were incubated with Alexa Fluor Plus 488 (A32731, Invitrogen) or Alexa Fluor Plus 555 (A32727, Invitrogen) secondary antibody for 1.5 h at room temperature. After one wash with 0.5% Triton X-100 and 8% sucrose in 1× PBS, DNA was stained with DAPI/Hoechst (1:1) (D9542, Sigma–Aldrich and B2261, Sigma–Aldrich, respectively) for 15 min at room temperature. After three washes with 1× PBS, chambers were removed, and slides were covered with coverslips using ProLong^™^ Glass Antifade Mountant (P36980, Invitrogen), sealed with nail polish 48 h later, and stored at −20°C for long-term storage.

### Fluorescence images acquisition and analysis

Fluorescence images were acquired at the Yale West Campus Imaging Core using the laser scanning confocal microscope Leica Stellaris 8 Falcon with the HC PL APO ×63/1.40 oil objective utilizing the Type F Immersion liquid (11944399, Leica Microsystems, Wetzlar, Germany). 405-nm laser was used for DAPI/Hoechst (cell nuclei staining) while White Light Laser (WLL) was used for Alexa Fluor Plus 488 (RAD51 or pRPA32 S4/8 staining) and Alexa Fluor Plus 555 (BRCA1 staining) excitations. HyD detectors were used for a subsequent fluorescence detection. Leica Application Suit X (LAS X) software (Leica Microsystems) was used for optimization of fluorescence detection, signal yields, and spectral unmixing. All images were taken without overexposure at the identical laser intensity, gain, and exposure parameters. The images were saved as 1024 pixels × 1024 pixels, 8-bit multi-channel Leica Image Files (.lif) and exported for quantification to 8-bit TIFF format files using Fiji software (NIH, Bethesda, MD, USA) [34]. RAD51, pRPA32 S4/8, and BRCA1 foci were quantified using Focinator software [35]. Representative images for figures were prepared using Fiji with QuickFigures plugin [36] and exported to Adobe Illustrator software (Adobe, Mountain View, CA, USA) at 300 ppi. Brightness was linearly adjusted for a given fluorochrome uniformly across entire images and figures to enhance visibility while ensuring the data are accurately represented.

### Statistical analyses

All analyses were performed using GraphPad Prism Software. Data are presented as mean ± SEM and were analyzed by two-way ANOVA with a Tukey post hoc test; *n* = 3 independent experiments unless otherwise indicated. ****P < 0.0001, ***P < 0.001, **P < 0.01, *P < 0.05.

## Results

### PARPi-resistant IDH1-mutant tumors exhibit end protection factor suppression

**Figure 1.**
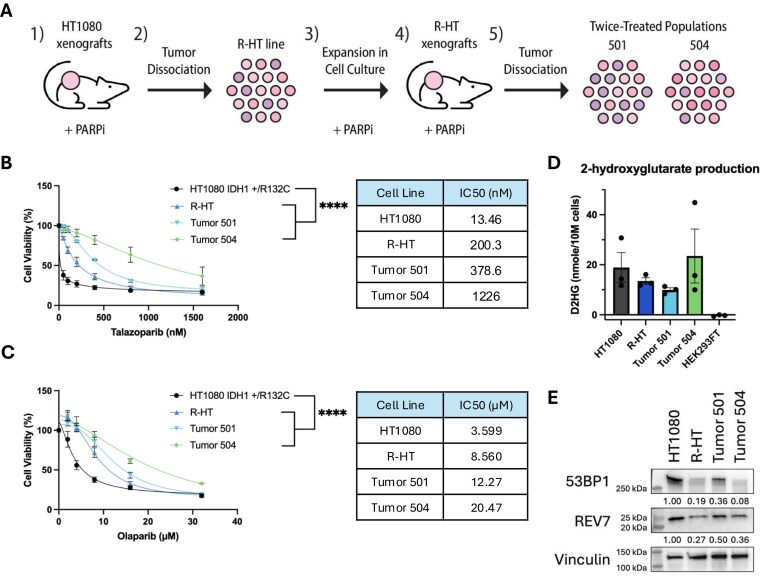
PARPi-resistant tumors selected *in vivo* show downregulation of end protection factors 53BP1 and REV7. (**A**) Schematic of serial tumor xenograft approach to generate resistant tumor cell populations using the PARPi talazoparib. An initial tumor xenograft study with the patient-derived IDH1-mutant fibrosarcoma cell line HT1080 was conducted, and the three largest PARPi-resistant tumors were dissociated, combined, and expanded in culture. A second xenograft study with this PARPi-resistant population (R-HT) was conducted, and the two largest PARPi-resistant tumors (Tumor 501 and Tumor 504) were dissociated and expanded in culture. These twice-treated bulk tumors were maintained in culture with 25 nM talazoparib treatment. (**B**) Evaluation of short-term cell viability via CellTiter-Glo luminescence assay 6 days after treatment with increasing concentrations of the PARPi talazoparib in HT1080 twice-treated PARPi tumors. Talazoparib IC50 of tumor-derived cell lines compared to parental HT1080 cell line. IC50s were calculated using non-linear regressions from the curves in Fig. 1B. Data are presented as mean ± SEM and were analyzed by two-way ANOVA; n = 3 independent experiments; ****P < 0.0001. (**C**) Evaluation of cell viability via CellTiter-Glo luminescence assay 6 days after treatment with increasing concentrations of the PARPi olaparib in HT1080 twice-treated PARPi tumors. Olaparib IC50 of tumor-derived cell lines compared to parental HT1080 cell line. IC50s were calculated using non-linear regressions from the curves in Fig. 1C. Data are presented as mean ± SEM and were analyzed by two-way ANOVA; n = 3 independent experiments; ****P < 0.0001. (**D**) D-2HG production in PARPi-resistant tumors as measured by the D-2HG enzyme assay. (**E**) Western blot analysis showing downregulation of end protection factors 53BP1 and REV7 in PARPi-resistant tumors. Vinculin was used as loading control.

We pursued an *in vivo* method for generating IDH-mutant tumors with acquired PARPi resistance: serial xenograft tumor studies utilizing HT1080 cells, a fibrosarcoma line carrying an endogenous *IDH1* R132C/+ mutation (see schematic in Fig. 1A). The tumor protocol consisted of two arms: vehicle versus talazoparib alone. Parental HT1080 tumors were mostly sensitive to talazoparib, yielding significant growth delay for some responder tumors. However, there was some heterogeneity, with other tumors showing lesser response (Supplementary Fig. S1A). These tumors reflect the variable responses expected to emerge in patients with IDH-mutant cancers undergoing PARPi treatment, motivating our strategy to evaluate the apparently resistant tumor populations.

At the completion of this initial round of tumor growth, we dissociated the three largest tumors that had grown out after the treatment course with the PARPi talazoparib and pooled these tumor cells to establish a population of resistant tumor cells. This first pooled tumor population is referred to as “R-HT.” We continued to treat these R-HT cells with talazoparib (25 nM) in cell culture and expanded the population to perform a second mouse xenograft tumor study. Mice were implanted with the R-HT pooled cell populations that had been expanded in the presence of talazoparib, and a second tumor growth study was carried out. As expected, R-HT tumors showed reduced response to talazoparib treatment in the second xenograft study (Supplementary Fig. S1B). From this second study, we again selected and dissociated the two largest tumors in talazoparib-treated mice (mouse 501 and mouse 504) and established them as separate bulk tumor populations in cell culture, here on referred to as “twice-treated tumor” populations. We tested these cell populations for sensitivity to PARPi in short-term cell viability assays. Treatment with talazoparib and another PARPi, olaparib, revealed PARPi resistance in R-HT cells, as well as in the twice-treated tumor populations (501 and 504) (Fig. 1B and C). These results demonstrate the R-HT population’s *in vivo* PARPi resistance and present our twice-treated tumor populations 501 and 504 as robustly resistant models. Through this approach, we generated a patient-relevant resistance model, utilizing cells with an endogenous IDH1 mutation and leveraging *in vivo* PARPi treatment.

Reversion mutations are a common mechanism of PARPi resistance clinically in BRCA-deficient cancers, particularly small intragenic deletions that restore the reading frame in BRCA1/2 [37–39]. Following a similar hypothesis, we sought to determine if the IDH1 mutation was still present in these HT1080 PARPi-resistant tumors by measuring the levels of intracellular D-2HG in the resistant tumors as a read-out for the presence of the IDH1 mutation. As shown in Fig. 1D, although D-2HG levels vary between tumors populations, they are all still elevated relative to HEK293FT cells, a non 2HG-producing cell line that is IDH1 WT. The data suggest reversion of the IDH1 mutation is not the driver of PARPi resistance in the resistant tumor populations.

Next, we sought to investigate other mechanisms of PARPi resistance, focusing on PARPi resistance pathways previously described in the context of BRCA1/2-mutant cancers. Loss of end protection factors, 53BP1 and REV7, and members of the Shieldin complex have been shown to lead to PARPi resistance in BRCA1-mutated cancers via restoration of HDR [18–20, 24, 25, 28]. Upon damage, 53BP1 is localized to the site of DSBs and recruits downstream effectors including REV7, protecting the damaged DNA ends from resection [40–43]. In BRCA1-mutated cancers, loss of these end protection factors restores HDR by allowing for resection of the damaged DNA ends to enable HDR.

We assessed expression levels of these negative regulators of HDR in the initial pooled R-HT PARPi-resistant population and the twice-treated bulk populations from the 501 and 504 tumors from the second tumor growth study. The initial survey via western blot analyses revealed substantial downregulation of end protection factors 53BP1 and REV7 across all three tumor populations to varying levels (Fig. 1E). The extent of downregulation across PARPi-treated tumor populations provided early evidence for the importance of loss of end protection in the IDH1-mutant context and further supports the notion that the PARPi sensitivity in IDH-mutant cancers is a consequence of a defect in HDR.

### Depletion of 53BP1 and REV7 confers PARPi resistance *in vitro*

**Figure 2.**
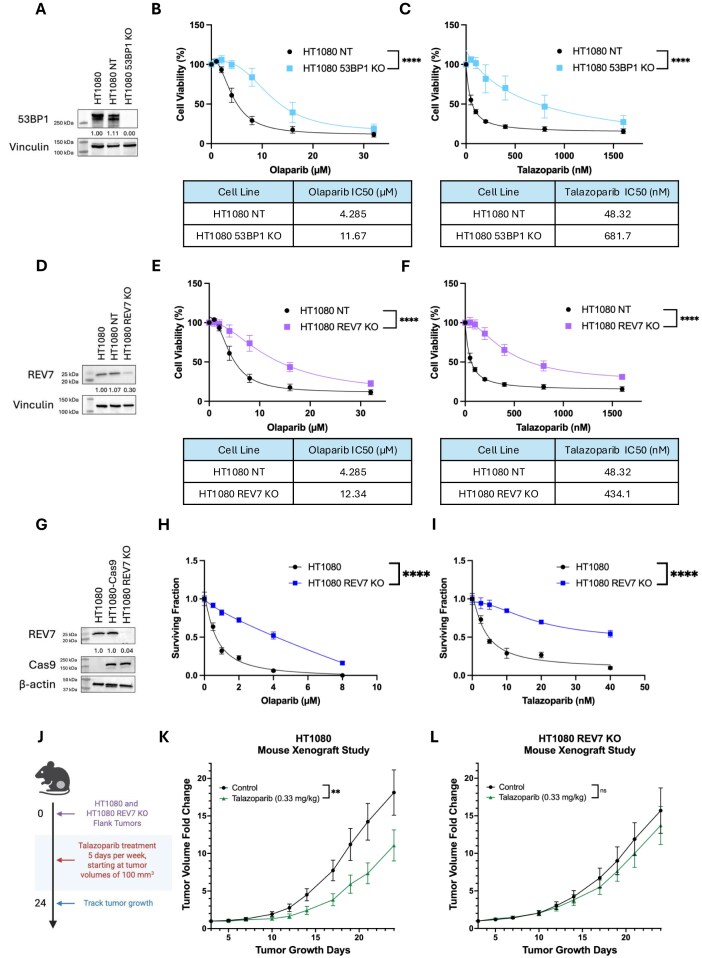
Depletion of 53BP1 and REV7 in HT1080 IDH1 R132C/+ cell line leads to PARPi resistance *in vitro* and *in vivo*. (**A**) Western blot analysis showing CRISPR–Cas9-mediated knockout of 53BP1 in HT1080 cells compared to parental HT1080 and a non-targeting control (HT1080 NT). (**B** and **C**) Evaluation of short-term cell viability via CellTiter-Glo luminescence assay 6 days after treatment with increasing concentrations of the PARPi olaparib (B) and talazoparib (C) in HT1080 53BP1 knockout cells compared to HT1080 NT control. IC50 values were calculated using nonlinear regressions from the curves in Fig. 2B and C. Data are presented as mean ± SEM and were analyzed by two-way ANOVA; n = 3 independent experiments; ****P < 0.0001. (**D**) Western blot analysis showing depletion of REV7 in HT1080 knockout cell line. (**E** and **F**) Evaluation of short-term cell viability via CellTiter-Glo luminescence assay 6 days after treatment with increasing concentrations of the PARPi olaparib (E) and talazoparib (F) in HT1080 REV7 knockout cells compared to HT1080 NT control. IC50 values were calculated using non-linear regressions from the curves in Fig. 2E and F. Data are presented as mean ± SEM and were analyzed by two-way ANOVA; n = 3 independent experiments; ****P < 0.0001. (**G**) Western blot analysis showing depletion of REV7 using a Cas9-expressing HT1080 cell line. (**H** and **I**) Long-term clonogenic survival of HT1080 and HT1080 REV7 KO lines treated with increasing doses of PARPi olaparib (H) and talazoparib (I). Data are presented as mean ± SEM and were analyzed by two-way ANOVA; n = 3 independent experiments; ****P < 0.0001. (**J**) Schematic of tumor growth delay experiment in HT1080 REV7 +/+ and HT1080 REV7 KO cells *in vivo*. Mice were treated via oral gavage with talazoparib (0.33 mg/kg) 5 days per week, beginning at tumor volumes of 100 mm^3^. Created in BioRender. Colon Rios, D. (2025) https://BioRender.com/q9nhipo. (**K** and **L**) Growth curves of HT1080 REV7 +/+ (K) and HT1080 REV7 KO (L) xenograft tumors in untreated mice and in mice treated with talazoparib (two-way ANOVA, control versus talazoparib: HT1080 interaction **P = 0.0092, REV7 KO interaction *P* = 0.9926; *n* = 8 mice).

Given that these PARPi-resistant populations are heterogenous in nature, we sought to probe the effects that specific loss of the end protection factors 53BP1 and REV7 might have on IDH1-mutant cancer cells. For this, we utilized Cas9 ribonucleoprotein and sgRNAs to target 53BP1 in HT1080 cells to generate 53BP1 knockout HT1080 cells (Fig. 2A and Supplementary Fig. S2A). Upon treatment of these cells with two PARPi, olaparib and talazoparib, we found that 53BP1 knockout in the HT1080 cell line resulted in substantial resistance to both PARPi as measured by short-term cell viability assays (Fig. 2B and C). Likewise, we used Cas9 ribonucleoprotein and sgRNAs to target REV7 expression in HT1080 (Fig. 2D and Supplementary Fig. S2B). Unlike the HT1080 53BP1 knockout cell line, a complete knockout of REV7 was not achieved using this method. REV7 is essential for cellular processes such as mitosis and genomic maintenance [44]. Given the increased replication stress in IDH-mutant cancers [9], it is possible that complete knockout in this context is lethal to these cells. Nonetheless, knockout of 70% REV7 expression was sufficient to yield cells resistant to PARPi treatment (Fig. 2E and F).

To further investigate if loss of REV7 leads to resistance *in vivo*, we generated an additional HT1080 knockout line for REV7 using the established lentiGuide-Puro/lentiCas9-Blast system [33]. CRISPR–Cas9-expressing HT1080 cells were selected by blasticidin resistance, grown up as single-cell colonies and screened for CRISPR–Cas9 protein expression via western blot analysis (Fig. 2G and Supplementary Fig. S2C). REV7 sgRNAs were then introduced into CRISPR–Cas9-expressing HT1080 cells using the lentiGuide-Puro lentiviral vector, with cells expressing both vectors gaining blasticidin and puromycin resistance. Single-cell colonies were grown up and screened for knockout via western blot analysis (Fig. 2G). In this case, there was almost complete loss of REV7 expression, leading to clear resistance to both olaparib and talazoparib *in vitro* as measured in a long-term cell viability assay (Fig. 2H and I).

We sought to determine if this loss of REV7 could drive resistance in IDH1-mutant tumors. We assessed differences between the parental HT1080 and HT1080 REV7 KO lines in tumor growth delay assays in mice treated with talazoparib, compared to a vehicle-treated control arm (Fig. 2J). Significant tumor growth delay from talazoparib treatment was seen with parental HT1080 tumors (Fig. 2K), consistent with prior work, but this effect was lost in REV7 KO tumors (HT1080, *P* = 0.0092 versus REV7 KO, *P* = 0.9926) (Fig. 2L). This clear difference in treatment response provides evidence for the impact of REV7 loss on PARPi resistance in the IDH-mutant context.

### Loss of end protection factors in IDH1-mutant cells restores HDR

**Figure 3.**
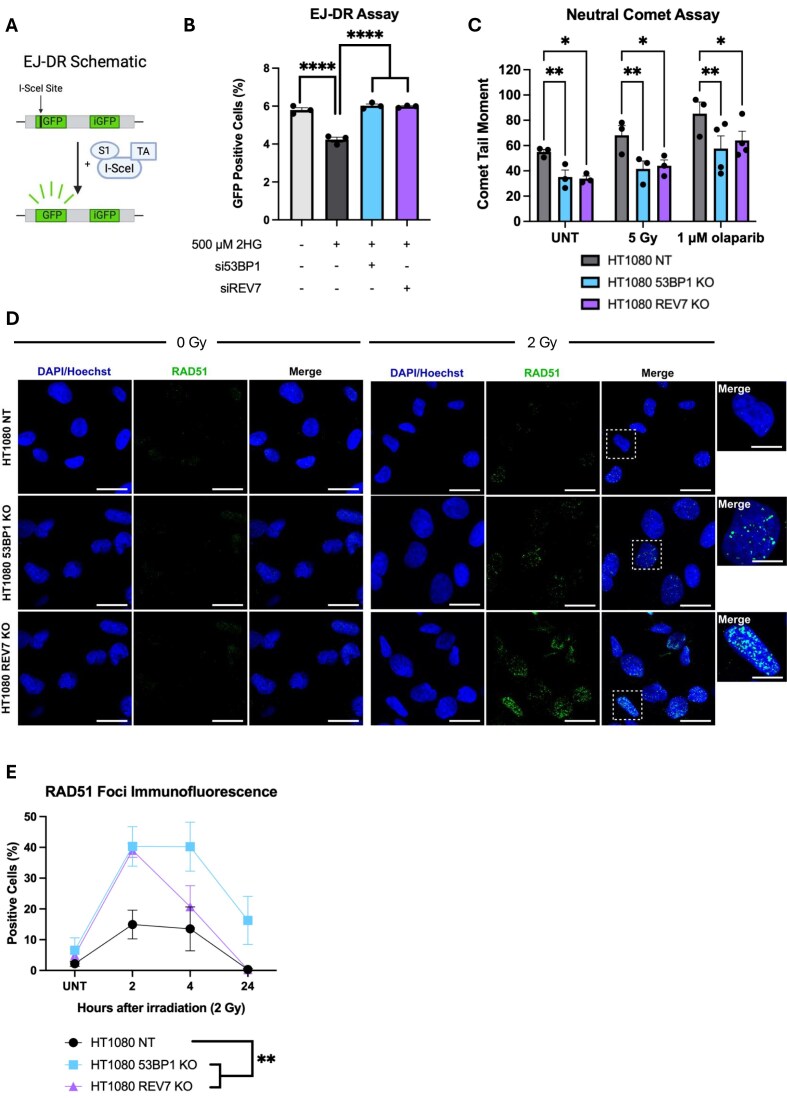
Knockout of either 53BP1 or REV7 decreases DNA damage and restores HDR. (**A**) Schematic of the EJ-DR chromosomally based reporter assay to measure HDR efficiency. Shield-1 (S1) and triamcinolone acetonide (TA) stabilize I-SceI and induce nuclear translocation. Created in BioRender. Colon Rios, D. (2025) https://BioRender.com/s775wjw. (**B**) Quantification of HDR using the EJ-DR reporter assay. Data are presented as mean ± SEM and were analyzed by ordinary one-way ANOVA with a Tukey post hoc test, ****P < 0.0001, *n* = 3 technical replicates. (**C**) Characterization of baseline DNA damage, radiation-induced damage and PARPi-induced damage in HT1080 knockout cell lines as measured by neutral comet assay 4 h after treatment with 5 Gy, or 24 h after treatment with 1 µM olaparib. Data are presented as mean ± SEM and were analyzed by two-way ANOVA with a Tukey post hoc test; n = 3 independent experiments; **P < 0.01, *P < 0.05. (**D**) Representative images of RAD51 foci in HT1080 and knockout cell lines at baseline and 2 h post irradiation with 2 Gy (green, RAD51; blue, DAPI/Hoechst; scale bars are 25 µm; insets are zoomed 2.5× and scale bars are 10 µm). (**E**) Percentage of RAD51 positive cells (threshold: 10 foci/nuclei) of HT1080 NT, HT1080 53BP1 KO, and HT1080 REV7 KO at indicated times post ionizing radiation (2 Gy). Data are presented as mean ± SEM and were analyzed by two-way ANOVA with a Tukey post hoc test; **P < 0.01; *n* = 3 independent biological replicates, at least 500 nuclei analyzed.

To begin examining the role of 53BP1 and REV7 in restoration of HDR, we utilized the EJ-DR reporter assay that measures HDR capacity (Fig. 3A). U2OS EJ-DR cells are a human osteosarcoma cell line with the DR-GFP chromosomally integrated green florescent protein (GFP)-based HDR reporter [45]. The assay involves creating a DSB by a ligand-inducible I-SceI site-specific nuclease that cuts a defined site within the upstream GFP gene in the chromosomally integrated GFP reporter. HDR activity at the induced DSB reconstitutes a functional GFP gene by recombination between the two GFP gene fragments. Although the U2OS cell line does not harbor IDH mutations, the addition of 2HG to the medium of the cells is able to phenocopy the presence of the mutation, as shown previously by our group and others [2, 3, 8]. Using this approach, we confirmed our previous result that exposure of the U2OS EJ-DR cells to 2HG suppresses HDR following I-SceI induction [46], as measured by the proportion of GFP-positive cells via flow cytometry (Fig. 3B). However, we found that cells pretreated with siRNAs targeting either 53BP1 or REV7 show reversal of the HDR-deficiency caused by 2HG (Fig. 3B and Supplementary Fig. S3A).

Our group and others have also shown that IDH-mutant tumor cells have increased baseline DNA damage compared to IDH-wild-type cells, as measured by the comet assay, reflecting the defect in HDR. This increased DNA damage burden is present both in untreated cells and upon treatment with DNA damaging agents [2, 47]. We wanted to determine if restored HDR due to depletion of end protection factors would correlate with decreased DNA damage. We evaluated the effect that depletion of these proteins has on constitutive (baseline) DNA damage, ionizing radiation-induced damage, and PARPi-induced damage via neutral comet assay (Fig. 3C). At baseline, depletion of either 53BP1 or REV7 reduces the amount of damage in these cells, as indicated by shorter comet tail moments. Furthermore, similar effects were observed upon treatment with 5 Gy ionizing radiation and the PARPi olaparib, where the comet tails in the HT1080 knockouts were again lower than in parental HT1080 cells. These results show that loss of 53BP1 or REV7 in HT1080 cells reduces the levels of DNA damage, consistent with restoration of HDR capacity and increased repair of DSBs that may occur constitutively through endogenous processes (such as collapsed replication forks) or exogenous sources such as ionizing radiation and PARPi treatment.

RAD51 foci formation is another endpoint used to measure the ability of cells to undergo HDR, as loading of RAD51 onto single-stranded DNA by BRCA2 initiates strand invasion to facilitate recombination. To further explore restoration of HDR as a mechanism of PARPi resistance, we investigated the ability of HT1080 parental cells versus the 53BP1 and REV7 knockout subclones to form RAD51 foci after ionizing radiation. As shown in Fig. 3D and E, loss of 53BP1 or REV7 resulted in the restoration of RAD51 foci formation after DNA damage, as 2 h after treatment with ionizing radiation, 53BP1 and REV7 KO had more RAD51-positive cells. These results suggest that loss of end protection factors in IDH1-mutant cells may lead to PARPi resistance via restoration of HDR, as shown by increased HDR efficiency, decreased DNA damage burden, and increased RAD51 foci formation.

We also found that 53BP1 KO and REV7 KO in HT1080 cells led to increased BRCA1 foci-positive cells when compared to the nontargeting HT1080 control (Supplementary Fig. S3B and D), again suggesting increased localization of HDR factors to the site of damage and supporting our model of increased HDR activation upon loss of end-protection factors. We further observed small increases in pRPA32 S4/8-positive cells in the 53BP1 and REV7 knockout lines when compared to the HT1080 non-targeting control (Supplementary Fig. S3C and D), but these differences were not statistically significant. However, given the role of pRPA32 S4/8 in the replication stress response [48], it is plausible that the replication stress induced by ionizing radiation treatment serves as a confounding factor when studying end resection in isolation. Nonethless, the above data support our model of increased localization and HDR factors and reactivation of HDR upon loss of end protection factors 53BP1 and REV7 in IDH1-mutant cancers.

### Cediranib suppresses HDR and resensitizes PARPi-resistant IDH1-mutant cells to PARPi treatment

Cediranib is a receptor tyrosine kinase inhibitor that was developed to inhibit vascular endothelial growth factor receptor (VEGFR) to suppress angiogenesis in tumors [49]. An early clinical trial evaluated the combinatorial effects of cediranib and PARPi in patients with recurrent platinum-sensitive ovarian cancers [50]. The clinical trial revealed that a subset of patients without documented BRCA1/2 mutations showed increased progression free survival and overall survival. Mechanistic work from our group revealed that cediranib can suppress HDR gene expression through an E2F4-dependent mechanism [32], conferring sensitivity to PARPi. Specifically, cediranib treatment induces the formation of p130/E2F4 complexes that bind to E2F consensus sequences in the promoters of HDR genes, reducing gene expression. This is through the inhibition of PDGFRβ, which it also inhibits with nanomolar potency [32], causing activation of phosphatase PP2A, dephosphorylation of p130, and formation of repressive E2F4/p130 complexes, leading to transcriptional downregulation of BRCA1, BRCA2, and RAD51. A randomized trial by Kim *et al.* subsequently extended these findings into the clinic in prostate cancer patients, comparing olaparib treatment with or without cediranib in metastatic castration-resistant prostate cancer. The results showed improved progression free survival in the cediranib/olaparib combination arm compared to the olaparib alone arm [51].

**Figure 4.**
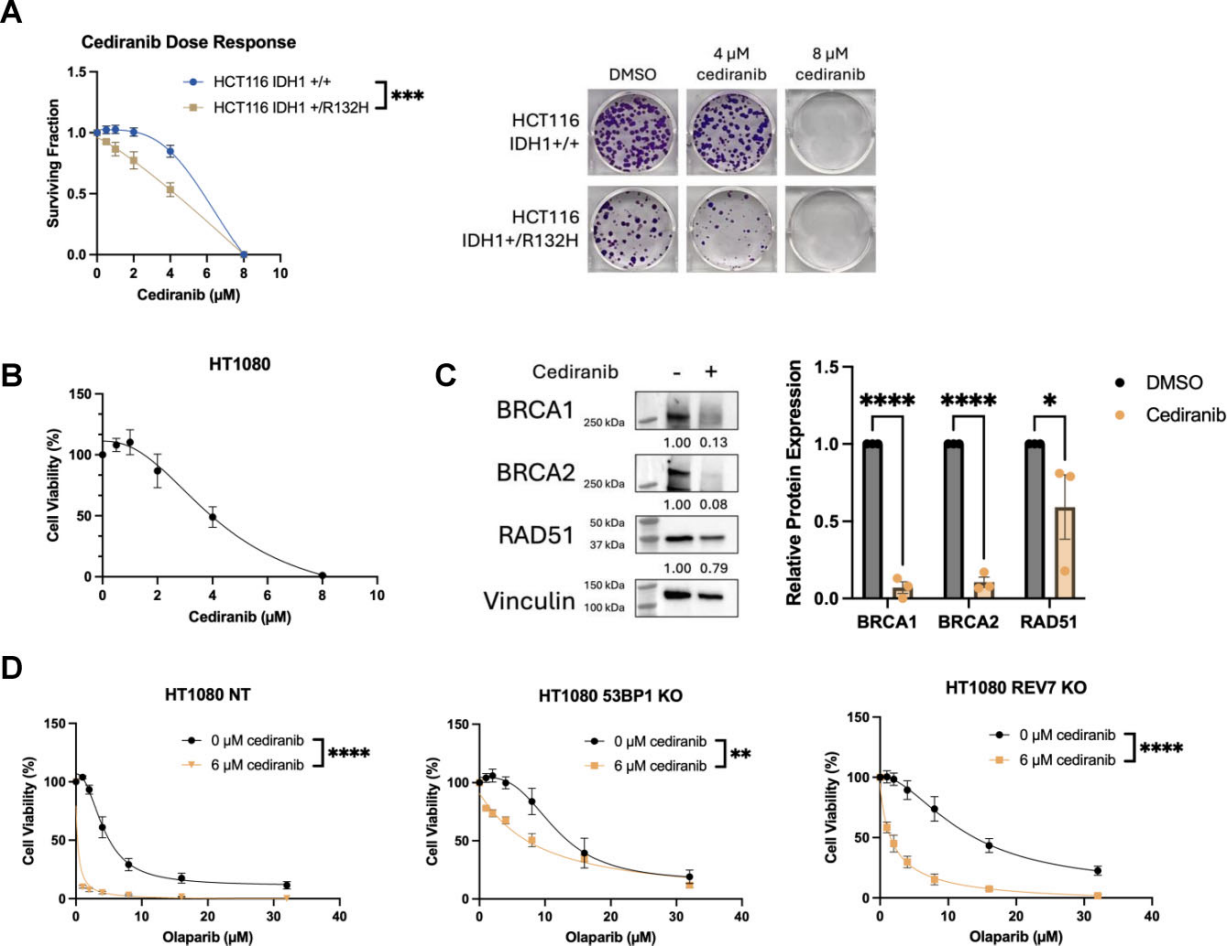
Cediranib treatment overcomes PARPi resistance in 53BP1 and REV7 knockout cell lines via suppression of HDR. (**A**) Evaluation of long-term cell viability via clonogenic survival assay 14 days after treatment with increasing concentrations of cediranib in HCT116 IDH1 +/+ versus HCT116 IDH1 +/R132H mutant cells. Representative images of colony formation are also shown. (**B**) Evaluation of short-term cell viability via CellTiter-Glo luminescence assay 6 days after treatment with increasing concentrations of cediranib in HT1080 IDH1 +/R132C cells. (**C**) Western blot analysis showing downregulation of HDR factors BRCA1, BRCA2, and RAD51 in HT1080 cells treated with 10 µM cediranib for 48 h. Vinculin was used as loading control. (**D**) Evaluation of short-term cell viability via CellTiter-Glo luminescence assay 6 days after treatment with increasing concentrations of the PARPi olaparib in combination with 6 µM cediranib in control HT1080 NT cells or HT1080 cells with either 53BP1 or REV7 knockout. Data are presented as mean ± SEM and were analyzed by two-way ANOVA with a Šidák post hoc test; *n* = 3 independent experiments; ****P < 0.0001, ***P < 0.001, **P < 0.01, *P < 0.05.

The HDR defect in IDH1-mutant cells is at the point of chromatin signaling and DNA repair factor recruitment to sites of damage, but the DNA repair factors are expressed at normal levels [3]. As discussed above, cediranib acts on HDR at a different point by suppressing expression of downstream factors like BRCA1, BRCA2, and RAD51 [32]. Hence, we asked if the HDR defect present in IDH-mutant tumors would render these cells sensitive to further suppression of DNA repair by an orthogonal mechanism via cediranib treatment. For this, we utilized the HCT116 colorectal carcinoma cell line with and without a CRISPR-engineered knock-in IDH1 R132H mutation (HCT116 IDH1 +/R132H). We found that HCT116 IDH1 +/R132H cells were more sensitive to cediranib treatment when compared to their wild-type counterpart, as measured by long-term clonogenic growth (Fig. 4A), indicating a synthetic lethal vulnerability. Treatment with cediranib was also effective against HT1080 cells, further supporting a synthetic lethal effect of cediranib in tumors cells harboring IDH mutations (Fig. 4B). Western blot analysis of cells 48 h after treatment with cediranib revealed decreases in BRCA1, BRCA2, and RAD51 expression (Fig. 4C, with replicates shown in Supplementary Fig. S4), consistent with prior work in multiple other cancer cell lines [32], and supporting the proposed mechanism of synthetic lethality.

This prompted us to test the potential efficacy of combining cediranib and olaparib treatment in the HT1080 and the HT1080 PARPi-resistant 53BP1 and REV7 knockout sub-lines. Short-term cell viability assays revealed that combination treatment with cediranib not only yielded increased sensitivity to olaparib treatment in the nontargeting control HT1080 cells but also in the 53BP1 and REV7-depleted HT1080 sub-lines, overcoming the resistance to the PARPi (Fig. 4D). Taken together, the above results suggest that cediranib can resensitize PARPi-resistant tumor cells to PARPi treatment and may also be effective in combination in IDH1-mutant cells even before resistance arises.

## Discussion

The work reported here provides evidence that loss of end protection factors in IDH1-mutant cancers leads to PARPi resistance both *in vitro* and *in vivo*. A serial transplantation xenograft study using a patient-derived cell line with an endogenous IDH1 mutation to enrich for PARPi-resistant cells revealed downregulation of end protection factors 53BP1 and REV7. We validated this resistance phenotype by creating 53BP1 and REV7 knockouts in the parental IDH1-mutant tumor cells and by further characterizing the effects on the DNA damage response. Our data suggest that loss of 53BP1 and REV7 restores the ability of these cells to carry out HDR at DSBs, leading to an overall decrease in DNA damage burden. Moreover, we demonstrate that IDH1-mutant tumors have a synthetic lethal response to the receptor tyrosine kinase inhibitor, cediranib, an agent previously shown to suppress DNA repair gene expression. In IDH1-mutant cells that have become resistant to PARPi via loss of 53BP1 or REV7 expression, we show treatment with cediranib resensitizes the cells to PARPi, providing insight into therapeutic regimens that can provide increased clinical benefit to patients.

Mechanistically, we find that loss of 53BP1 or REV7, two members of the DNA end protection pathway at DSBs, leads to PARPi resistance and restores HDR in IDH1-mutant cells. These observations are in congruence with studies showing loss of these factors conferring resistance in BRCA1-mutated cancers [21–25], supporting the original observation that elevated 2HG confers a fundamental HDR defect [2, 3]. 2HG and other oncometabolites impact the initiation of HDR at the level of chromatin signaling and HDR factor recruitment, while 53BP1 and REV7 act at a DSB to tilt the balance of repair pathways away from HDR and toward nonhomologous end joining. Hence, with loss of these end protection factors, the balance of repair pathways shifts back and enables the residual downstream HDR activity to resume in IDH-mutant cells.

One limitation of this study is that we conducted a candidate approach to elucidate potential mechanisms of PARPi resistance. In future work, employing CRISPR–Cas9 screening approaches to knockout or activate the expression of genes at a genome-wide level may provide additional insight into other factors that can contribute to PARPi resistance in the setting of IDH mutations.

Since the initial finding that IDH1/2 mutations induce an HDR-deficient phenotype, and therefore impaired repair of DSBs, multiple clinical trials such as NCT03749187, NCT03953898, NCT02576444, and NCT05406700 have begun evaluating the response of IDH-mutant cancers to PARPi monotherapy and combination therapy. The results from these and other studies have seen clinical benefit in patients treated with PARPi [29, 52, 53]. Nonetheless, some patients do not benefit from this treatment, highlighting the need for additional work to identify molecular or clinical predictive markers of benefit from PARPi. It is likely that there are both intrinsic and acquired mechanisms of PARPi resistance, and this study is an initial effort to begin to define mechanisms of PARPi resistance in IDH-mutant cancers. In addition, by identifying cediranib as another agent that is synthetically lethal to IDH1-mutant cancers, and that can resensitize these resistant cancer cells to PARPi, this work may also provide the basis for new therapeutic strategies for patients with oncometabolite-producing malignancies.

## Supplementary Material

zcaf047_Supplemental_Files

## Data Availability

The data underlying this article are available in the article and in its online supplementary material.

## Notes

Present address: Office of Development, Memorial Sloan Kettering Cancer Center, New York, NY 10017, United States
