# Supplementary material for: PARP inhibitor resistance in IDH1-mutant cancers due to loss of end protection factors, 53BP1 and REV7
Source: NAR Cancer. 2025 Dec 3;7(4):zcaf047. doi: 10.1093/narcan/zcaf047 (PMC12675010; doi:10.1093/narcan/zcaf047)
Supplement: zcaf047_Supplemental_Files [file zcaf047_supplemental_files.zip › Supplementary Figures Resubmission.pdf]

Fig. S1A

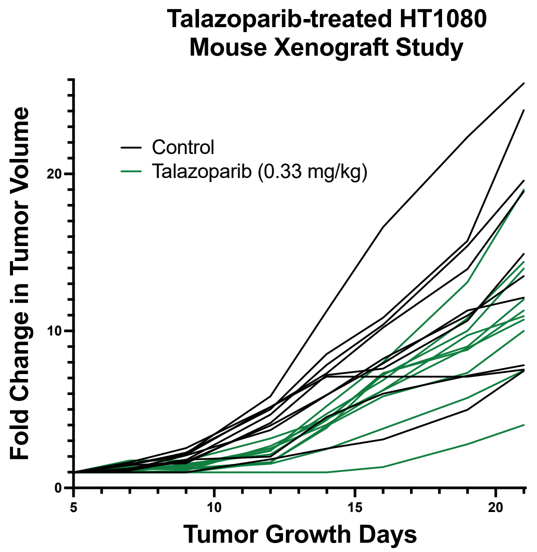

Fig. S1B

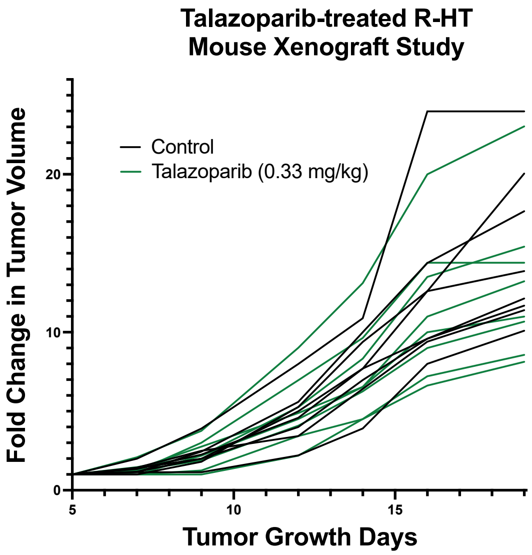

Supplementary Figure S1. A) Growth curves of HT1080 xenograft tumors in control mice and mice treated with talazoparib (0.33 mg/kg) (two-way ANOVA, control vs talazoparib, interaction P = 0.1765; n = 10 mice). B) Growth curves of R-HT xenograft tumors in control mice and mice treated with talazoparib (0.33 mg/kg (two-way ANOVA, control vs talazoparib, interaction P = 0.921; n = 8 mice)

Fig. S2A

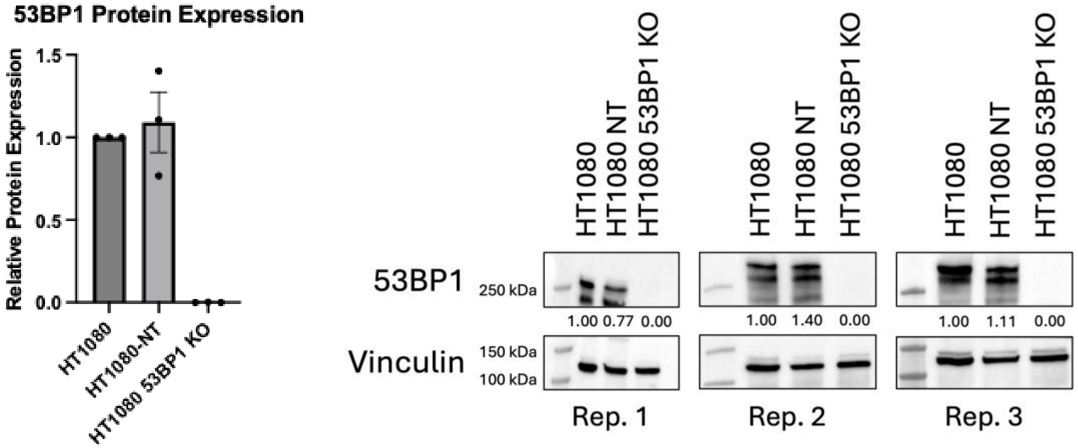

Fig. S2B

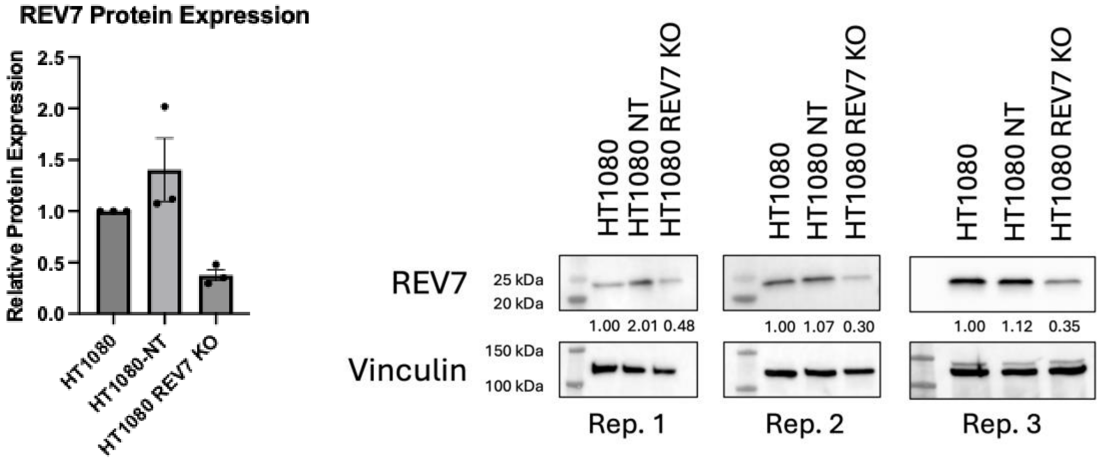

Fig. S2C

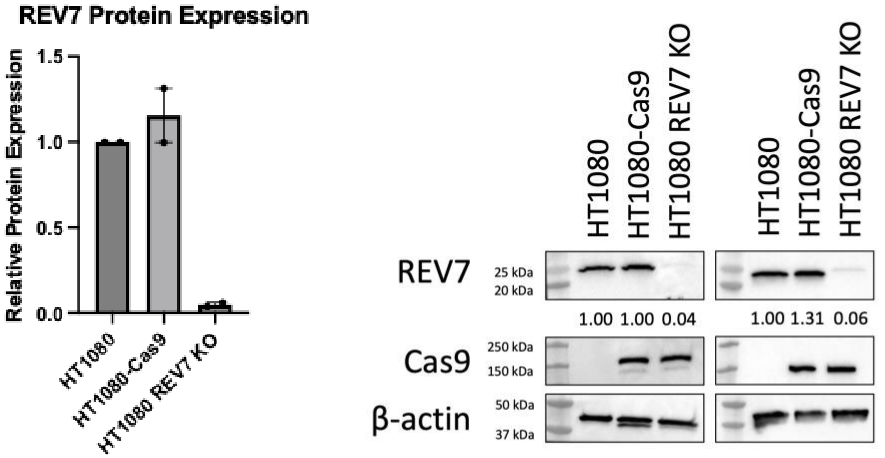

Supplementary Figure S2. A) Quantification and replicates of western blots showing 53BP1 KO. Vinculin was used as loading control. B) Quantification and replicates of western blots showing REV7 KO. Vinculin was used as loading control. C) Quantification and replicates of western blots showing REV7 KO using Cas9-expressing HT1080 cell line. β-actin was used as loading control.

Fig. S3A

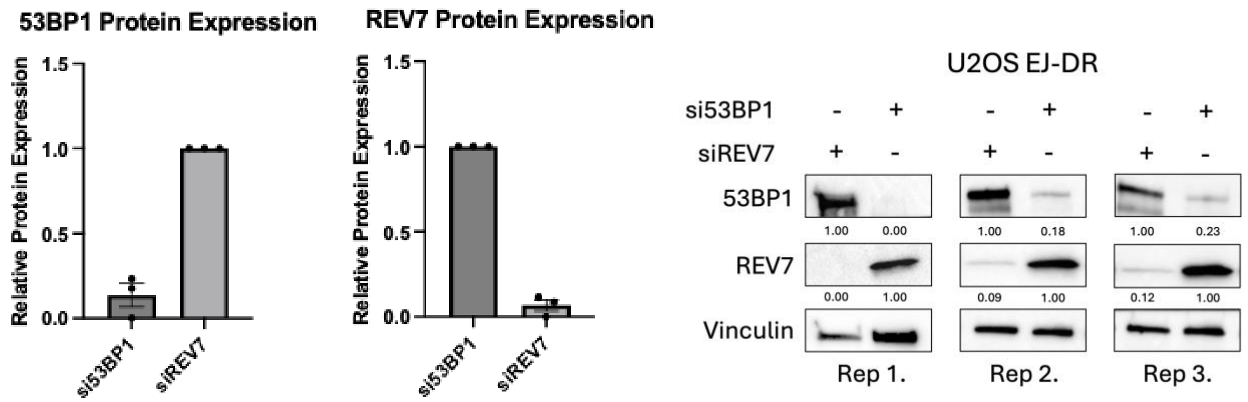

Fig. S3B

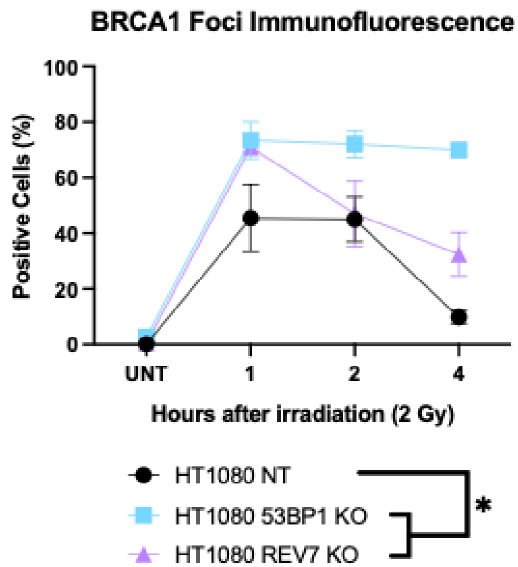

Fig. S3C

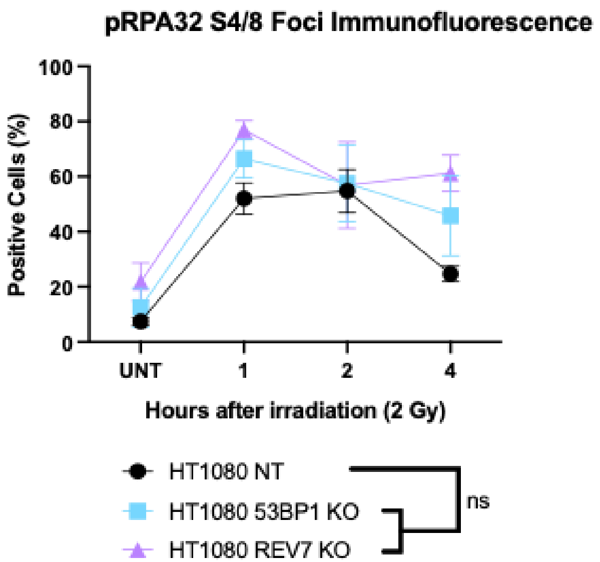

Fig. S3D

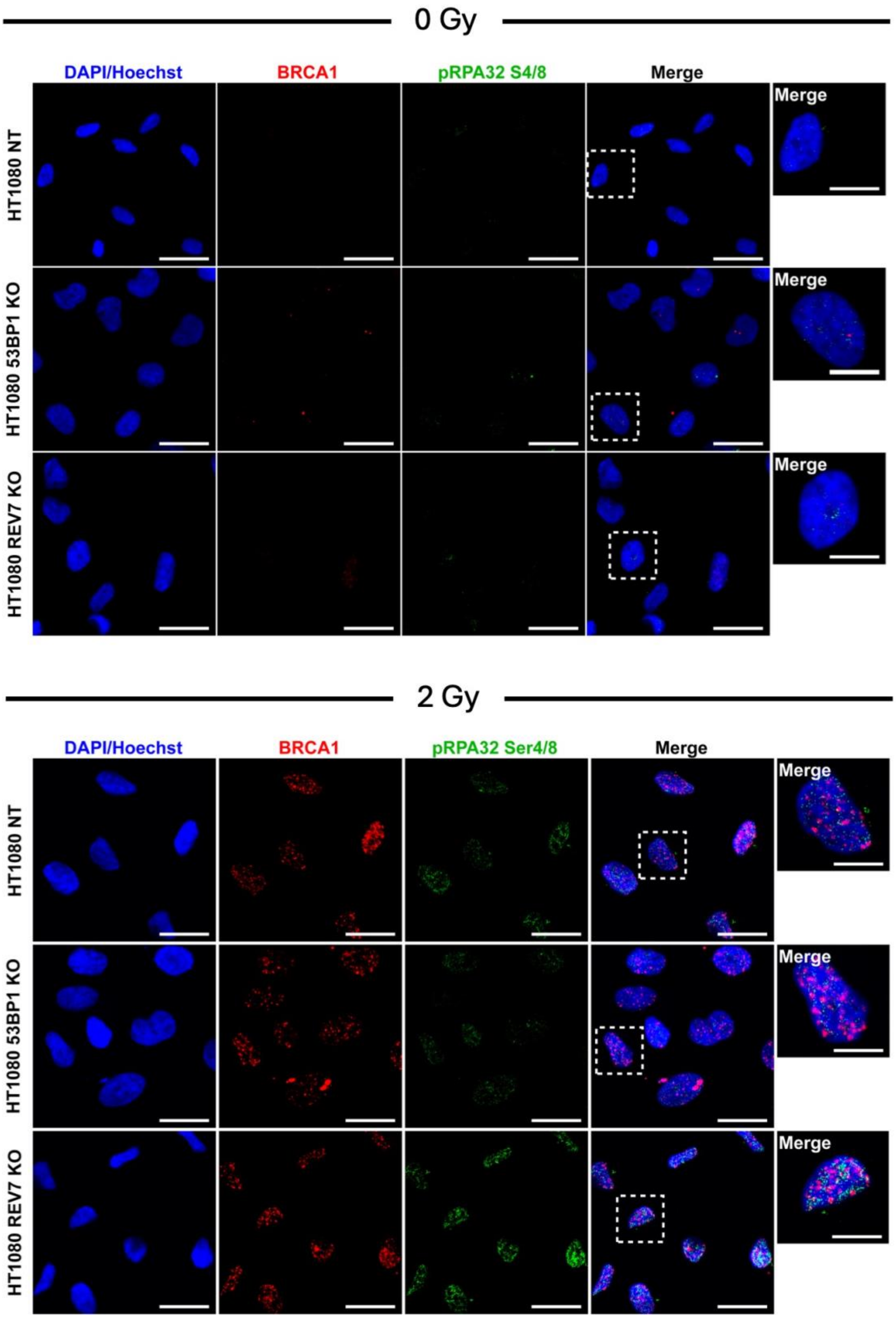

0 Gy
DAPI/Hoechst
BRCA1
pRPA32 S4/8
Merge
HT1080 NT
HT1080 53BP1 KO
HT1080 REV7 KO
Merge
2 Gy
DAPI/Hoechst
BRCA1
pRPA32 Ser4/8
Merge
HT1080 NT
HT1080 53BP1 KO
HT1080 REV7 KO
Merge

Supplementary Figure S3. A) Quantification and replicates of western blots for 53BP1 and REV7 in EJ-DR U2OS cells upon siRNA treatment. Vinculin was used as loading control. B) Percentage of BRCA1 positive cells (threshold: 10 foci/nuclei) of HT1080 NT, HT1080 53BP1 KO and HT1080 REV7 KO at indicated times post ionizing radiation (2 Gy). Data are presented as mean ± SEM and were analyzed by two-way ANOVA with a Tukey post hoc test; * $p < 0.05$; n = 3 independent biological replicates, at least 300 nuclei analyzed. C) Percentage of phospho-RPA32 (pRPA32 S4/8) positive cells (threshold: 10 foci/nuclei) of HT1080 NT, HT1080 53BP1 KO and HT1080 REV7 KO at indicated times post ionizing radiation (2 Gy). Data are presented as mean ± SEM and were analyzed by two-way ANOVA with a Tukey post hoc test; ns, n = 3 independent biological replicates, at least 300 nuclei analyzed. D) Representative images of BRCA1 and pRPA32 S4/8 foci in HT1080 and knockout cell lines at baseline and 1 hour post irradiation with 2 Gy (red, BRCA1; green, pRPA32 S4/8; blue, DAPI/Hoechst; scale bars are 25 µm; insets are zoomed 2.5x and scale bars are 10 µm).

Fig. S4

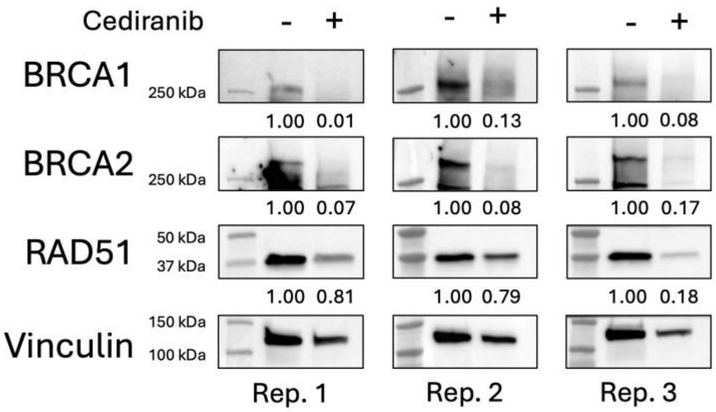

Supplementary Figure S4. Replicates of western blots for HDR factors BRCA1, BRCA2 and RAD51 in HT1080 cells upon treatment with 10 μM cediranib for 48 hours. Vinculin was used as loading control.
