# Supplementary material for: PARP inhibitor resistance in IDH1-mutant cancers due to loss of end protection factors, 53BP1 and REV7
Source: NAR Cancer. 2025 Dec 3;7(4):zcaf047. doi: 10.1093/narcan/zcaf047 (PMC12675010; doi:10.1093/narcan/zcaf047)
Supplement: zcaf047_Supplemental_Files [file zcaf047_supplemental_files.zip › Supplementary Table 1.pdf]

Table S1. List of single guide RNA (sgRNA) for generation of knockout cell lines.

Gene	sgRNA	Sequence
Non-targeting control	Edit-R Synthetic sgRNA Non-targeting Control #1	Cat No. U-009501-01-02
TP53BP1 (53BP1)	Edit-R Predesigned sgRNA SG-003548-01, TP53BP1	TGTGACAGCACAGCCCAGTA
	Edit-R Predesigned sgRNA SG-003548-02, TP53BP1	CTTTCAATCAAGGCCTTACT
	Edit-R Predesigned sgRNA SG-003548-03, TP53BP1	GGGTCCTTGACAGGACTAAT
MAD2L2 (REV7)	Edit-R Predesigned sgRNA SG-003272-01, MAD2L2	TATACTGATTCAGCTCCGGG
	Edit-R Predesigned sgRNA SG-003272-02, MAD2L2	TCCACTGCTGTCCATCAGGT
	Edit-R Predesigned sgRNA SG-003272-03, MAD2L2	AGAAGCCGCCACTCGCAACA
