# Supplementary material for: PARP inhibitor resistance in IDH1-mutant cancers due to loss of end protection factors, 53BP1 and REV7
Source: NAR Cancer. 2025 Dec 3;7(4):zcaf047. doi: 10.1093/narcan/zcaf047 (PMC12675010; doi:10.1093/narcan/zcaf047)
Supplement: zcaf047_Supplemental_Files [file zcaf047_supplemental_files.zip › Supplementary Table 2.pdf]

Table S2: List of primary antibodies used throughout the study.

Target	Dilution	Company	Catalog Number
53BP1	1:1000 in 5% BSA	Cell Signaling Technology	4937
β-actin	1:10000 in 5% Milk	Santa Cruz	sc-47778
BRCA1	1:500 in 5% Milk	Santa Cruz	sc-6954
BRCA2	1:5000 in 5% Milk	Millipore-Sigma	OP95
Cas9 *(S. pyogenes)*	1:1000 in 5% Milk	Cell Signaling Technology	14697
GAPDH-HRP	1:10000 in 5% Milk	Protein Tech	HRP-60004
MAD2L2 (REV7)	1:1000 in 5% Milk	Novus	NBP1-02798
RAD51	1:1000 in 5% Milk	Millipore-Sigma	PC130
Vinculin	1:5000 in 5% Milk	Abcam	ab129002
